# Critical care in developing nations: what has the COVID-19 pandemic revealed?

**DOI:** 10.11604/pamj.2023.45.123.40574

**Published:** 2023-07-14

**Authors:** Luiz Alberto Cerqueira Batista Filho

**Affiliations:** 1 Research Department, Imed Group, São Paulo, Brasil

**Keywords:** Developing countries, critical care, COVID-19, intensive care

## Abstract

The COVID-19 pandemic has altered the lives of millions of individuals, resulting in over 600 million cases and over 6 million fatalities worldwide. In developing nations, mortality rates for intubated patients with viral pneumonia were as high as 80%, compared to 30% in developed countries. This article intends to discuss the causes of this disparity, focusing on the main problems shared by nations with limited resources.

## Commentary

The ongoing COVID-19 pandemic has caused more than 600 million cases and over 6 million deaths globally [1]. There were repercussions in every aspect of human life, and the public health debate became highly politicized. During the pandemic, shortages of intensive care unit (ICU) beds, mechanical ventilators, and specialized healthcare employees plagued many nations, particularly in the world's poorest regions. It is now evident that low- and middle-income countries (LMICs) lack intensive care settings equipped to treat critically ill patients, as the mortality rate for severe COVID-19 pneumonia patients undergoing mechanical ventilation has reached up to 80% [2], compared to 30% in high-income countries (HICs) [3]. This astounding disparity underscores the need to discuss critical care as a matter of public health. ICUs will unquestionably play a larger role in the future as the population continues to age, not to mention the fact that they are crucial for the bed traffic of any hospital. After the pandemic, it should be a priority for LMICs to secure an adequate number of ICU beds as well as safer and more efficient intensive care facilities.

Insufficient data exist regarding the number of ICUs in developing nations [4]. It is nearly impossible to ascertain the precise number of ICUs in the majority of African nations, but it is reasonable to assume that the number is quite small, given the absence of electricity, internet, and even standard hospital beds. South and Central America, a number of Asian countries, and other impoverished regions of the world share a similar dearth of information. According to the World Health Organization, a ratio of between one and three ICU beds per ten thousand inhabitants is considered adequate, and many nations have attained this goal. However, it is difficult to determine the definition of an ICU bed based on numerous studies. The ratio of ICU units per 10,000 people in Nepal is estimated to be 2.80 [5]. However, 47 percent of these ICU patients lack mechanical ventilation. Taking into account beds with a mechanical ventilator at the bedside, the ratio of ICU beds in Nepal falls to 1.5 per 10,000 residents.

There is also a problem with the accessibility of ICUs to the general public, as not all of them are available to anyone. For example, Brazil has a national ratio of 2,2 ICU units per 10,000 residents [6]. However, these ICU spaces are divided between the public and private sectors. Private sector intensive care units are only accessible to the 23% of Brazilians with health insurance, as the remaining 77% rely on the public sector. Comparing the private and public sectors in terms of the number of ICU beds per 10,000 users reveals a significant disparity between the availability of ICU beds for Brazilians with and without health insurance. Local and global authorities should focus on these disparities in order to reduce healthcare access inequalities. Since the majority of critical care units are likely to be located in urban areas, it is reasonable to assume that many regions of these nations lack adequate critical care units, given that their ICU bed capacities are already inadequate.

As most ICUs are located in large cities, urban regionalization of critical care units is a problem for many nations. As a result, appropriate treatment is delayed, and the undesirable transportation of critically ill patients becomes the norm, leading to an increase in morbidity and mortality. During the COVID-19 pandemic, there were numerous reports of patients being transferred to other locations due to a lack of intensive care unit (ICU) beds in both developed and developing nations. As evidenced by the pandemic, local access to critical care is a genuinely valuable commodity.

The scarcity of specialized medical personnel is the most significant problem in critical care around the globe. Beginning in the 1980s, the specialized critical care physician emerged. Critical care is a relatively new specialty. Previously, anesthesiologists, surgeons, and clinicians were in command of intensive care units. In modern times, to become an intensivist, one must complete at least four years of specialty training following graduation from medical school. The profession is considered underpaid and a leader in exhaustion. It is uncommon for a medical student to aspire to become an intensivist, as most critical care physicians develop a preference for caring for the critically unwell after having a primary specialty. In the majority of medical institutions, intensive care is not a priority because medical students spend so little time studying critically ill patients.

Even in HICs, the lack of intensivists has been a problem for decades [7]. Numerous ICUs lack a critical care specialist, and as a result, they lack efficient protocols and multidisciplinary rounds, which are essential components of any good ICU. Absence of these professionals results in lost opportunities to treat time-sensitive critical conditions, such as pruning a patient with acute respiratory distress syndrome or administering fluid resuscitation for septic shock. As well as many other aspects of patient care, such as enteral nutrition, deep vein thrombosis prophylaxis, early mobilization, and medical reconciliation, the management of mechanical ventilation is inadequate. It is impossible to staff ICUs with only specialists in any nation, but employing non-specialists does not address the problem of insufficient critical care-trained physicians. Having specialized nurses and respiratory therapists is also essential, as they will be assisting patients directly the majority of the time. In developing countries, nurses with formal education in critical care are uncommon, and the majority of the nursing personnel in ICUs consists of nurse technicians, which is not an optimal solution in a critical care environment.

Monitoring is the hallmark of intensive care units. A significant portion of monitoring is performed by machines, which alert the multidisciplinary team whenever their sensors detect a potentially hazardous situation, such as low oxygen saturation in the blood or asynchronies between the patient and the mechanical ventilator. Nonetheless, human judgment remains crucial, either to interpret the alarms generated by monitors, infusion pumps, and mechanical ventilators, or to take immediate action to neutralize a recognized threat. In high-income countries such as Canada and the United States, the patient-to-nurse ratio in ICUs is one-to-one, which facilitates rapid recognition of problems with vasopressor infusion, secretions in the endotracheal tube, and sudden changes in vital signs. In LMICs, this ratio is approximately 1: 5 or lower. This reduces the ICU's capacity to prevent life-threatening adverse events, resulting in an increase in mortality and morbidity [8]. Unquestionably, this is a significant factor that contributes to the disparity between the mortality rates of intubated COVID-19 patients in LMICs and HICs. In the context of life-threatening diseases, surveillance is crucial, and a well-rounded nursing staff is essential.

In 2002, Hillman described “Critical Care Without Walls” for the first time in an editorial [9]. Good critical care necessitates the presence of intensivists in all areas of the hospital where they are required. A mobile, specialized critical care team aids in identifying potential ICU admission candidates and enables prompt intervention whenever clinical conditions deteriorate in hospitals or emergency rooms. This is the original concept behind the Fast Response Teams, which are essential to any tertiary healthcare facility. Obviously, the majority of hospitals in LMICs are a long way from this reality, but it is essential to keep this in mind as a future objective, particularly in developing nations.

The population is now much more aware of the importance of a decent ICU bed as a result of the COVID-19 outbreak. Many specialists believe that increasing the number of ICU beds is the way of the future, despite the fact that doing so would significantly increase the financial burden. Healthcare institutions and governments face difficulty in determining the appropriate quantity of critical care beds. After the traumatic pandemic experience, hospitals will likely implement flexible policies, increasing and decreasing the number of beds in response to demand [10]. However, COVID-19 has demonstrated that the most valuable resource for critical care is not technology, but rather human capital. Bringing cutting-edge devices to the bedside facilitates care for the critically ill, but without a specialized multidisciplinary team to operate them and, most importantly, care for patients, the mortality rate will not change. The pandemic's lessons should be rapidly assimilated so that the world is better prepared for the next outbreak.
